# Reconciling Coulter Counter and laser diffraction particle size analysis for aquaculture monitoring

**DOI:** 10.1007/s10661-024-12786-w

**Published:** 2024-06-28

**Authors:** T. G. Milligan, B. A. Law, V. Zions, P. S. Hill, K. Hua, C. W. McKindsey, A. Lacoursière‐Roussel

**Affiliations:** 1 Faculty of Graduate Studies, Dalhousie University, Halifax, NS Canada; 2 Fisheries and Oceans Canada, Bedford Institute of Oceanography, Dartmouth, NS Canada; 3 Department of Oceanography, Dalhousie University, Halifax, NS Canada; 4 Fisheries and Oceans Canada, Ottawa, ON Canada; 5 Fisheries and Oceans Canada, Institut Maurice Lamontange, Mont-Joli, QC Canada; 6 Fisheries and Oceans Canada, St Andrews Biological Station, St. Andrews, NB Canada

**Keywords:** Aquaculture monitoring, Coulter Counter, Reduced major axis, Grain size, Particle size, Laser diffraction, Sediment

## Abstract

The disaggregated inorganic grain size (DIGS) of bottom sediment analyzed with a Coulter Counter (CC) has been used to show that the fraction of sediment deposited in flocs (floc fraction) increased in both the near and far field after the introduction of open cage salmon aquaculture, altering benthic habitat and species composition. As a result, DIGS was identified as a potential indicator of regional environmental changes due to aquaculture. Laser diffraction is an attractive alternative to the CC because of its greater efficiency and larger size range. To determine if a laser diffraction instrument, Beckman-Coulter LS 13 320 (LS), could replace the CC within a Canadian national aquaculture monitoring program, the DIGS of 581 samples from five different regions in eastern Canada were analyzed with an LS and a CC. Results show that the LS could not be used to calculate floc fraction. Instead, % sortable silt and the volume % of inorganic particles < 16 µm were evaluated as alternative proxies for fine sediment properties. LS and CC values for these parameters were correlated, but they were significantly different and the relationship between the instruments was dependent on the area sampled. The LS did not capture variations between areas seen in the CC DIGS data. Where the DIGS from the CC found no sorting in the finest size classes, all the LS samples had similar size distributions characterized by smooth modal peaks. The LS and CC both return values that can be used to monitor changes in the deposition of fine-grained sediment, but the LS cannot determine changes in floc deposition and caution is required if comparing different sedimentary environments.

## Introduction

Analyzing the size distribution of the inorganic particles in sediment is a powerful approach for describing a location’s geomorphic setting and interpreting local fluid dynamics in the natural environment. It can be used to distinguish local versus regional sediment transport mechanisms and is a dominant controlling factor in sediment geochemistry. Since the introduction of electronic instruments to replace the traditional method of sieve and pipet (hydrometer) for sediment grain size analysis, there has been a concerted effort to determine how different methods compare and if the data can be used interchangeably (see Singer et al., 1988; Syvitski, 1991; Konert and Vandenberghe, 1997; Xu & Guida, 2003; Goosens, 2008).

Three different types of instruments for sediment grain size analysis have been the focus of most intercomparison studies: electro resistance particle sizing (e.g., Coulter Counter^©^, Elzone^©^), sedimentation (e.g., Sedigraph^©^), and laser diffraction, (e.g., Coulter LS^©^, Malvern MasterSizer^©^). Electro resistance particle sizing measures changes in impedance as a particle suspended in an electrolyte passes through an aperture of known diameter. Sedimentation determines particle size by measuring the change in concentration over time during settling and relating it to particle diameter using Stokes Law. Laser diffraction uses Fraunhofer or Mie theory to interpolate a size distribution based on the scattering pattern created by a laser passing through a suspension of particles. Of the three, only electro resistance measures the size of individual particles.

The development of a national Aquaculture Monitoring Program (AMP) by the Canadian Department of Fisheries and Oceans (DFO) was initiated in 2018. Its mandate is to assess the spatio-temporal interactions between aquaculture and the environment using consistent sampling and analysis methods across the country. In addition to benthic impacts at aquaculture sites there is concern for the long-term fate of aquaculture inputs and their transport to the far field. The disaggregated inorganic grain size (DIGS) of bottom sediment, which describes the size distribution of the inorganic particles after the removal of organic material, was proposed as a monitoring parameter. Aquaculture is not a source of inorganic sediment. Instead, the introduction of organic material from waste feed and feces can increase sedimentation of naturally occurring inorganic particles by enhancing flocculation (Milligan & Law, 2005). Flocculation is the term used for the process in aquatic environments that gathers colloids, organic material, and small inorganic particles to form loosely packed agglomerations of particulate material called flocs (Eisma, 1986; Kranck, 1973). Through flocculation, the settling velocity of fine-grained inorganic sediment and associated organic material can be increased by several orders of magnitude (Dyer & Manning, 1998; Fox et al., 2004; Law et al., 2014; Sternberg et al., 1999). The deposition of fine-grained sediment can negatively impact benthic habitats (Bilotta & Brazier, 2008; Pratt et al., 2014; Thrush et al., 2004). Flocculation is also a fundamental process governing the erosion, transport, and deposition of surface-active contaminants and their mobility can potentially alter the scale of the impacted zone (Zwolsman et al., 1996; Milligan & Loring, 1997; Kalnejais et al., 2007; Law et al., 2008; Milligan & Law, 2013; Little et al., 2015).

The DIGS of sediment, determined with a Coulter Counter, has been used to study sediment dynamics in several aquatic environments by determining floc fraction, the amount of material in a sediment that was deposited as flocs (Barry et al., 2013; Christiansen et al., 2000; Curran et al., 2004; deGelleke et al., 2013; Kranck, 1984; Law et al., 2013, 2019; Little et al., 2015; McCave & Hall, 2006; Milligan & Law, 2005; Milligan et al., 2007;). The principle behind using DIGS is that fine-grained inorganic particles are deposited within flocs or as single grains (Geyer et al., 2004; Kranck, 1980; Kranck & Milligan, 1985). The composition, size, density, and settling velocity of flocs can vary both temporally and spatially (Eisma, 1986; Kranck and Milligan, 1992; Mietta et al., 2009). Analyzing the DIGS avoids the need to study flocs in situ as bottom sediment integrates the transportation, deposition, and sorting of the inorganic particles (Kranck et al., 1996a, 1996b).

**Fig. 1:**
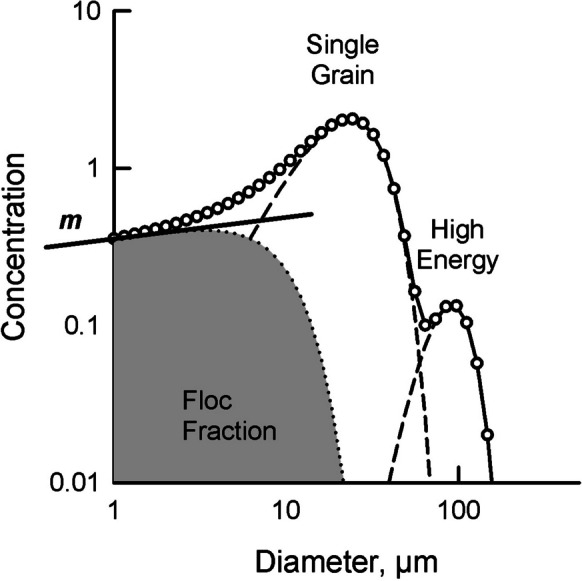
Idealized grain size distribution of the volume concentration of inorganic particles in a bottom sediment sample (circles) analyzed with a Coulter Counter showing floc fraction, the contribution from floc deposition (gray), and single grain settling (dashed line) determined with the model of Kranck and Milligan (1985) as modified by Curran et al. (2004). The high energy peak represents particles that can be introduced during high bottom stress events, possibly as bed load. *m* is the slope of the unsorted parent suspension predicted by Rosin and Rammler (1934) and Krumbein and Tisdel (1940), and the floc limit, d_f_ is the diameter at which the contribution of flocs and single grains to the bed is equal. Floc fraction and d_f_ vary with the amount of floc deposition

Weathering of rocks produces Rosin–Rammler size distributions which appear as straight lines when plotted as log concentration vs log diameter (Rosin & Rammler, 1934; Krumbein and Tisdel, 1940; Kranck & Milligan, 1985; Kranck & Milligan, 1991). These straight-line, unsorted, size distributions are modified during settling and subsequent resuspension (Kranck et al., 1996a, b). In aquatic environments, flocculation preserves the unsorted portion within the DIGS because flocculation gathers particles in the same proportions in which they occur in suspension (Khelifa & Hill, 2006; Kranck, 1980). Particles too large to be incorporated into flocs settle as single grains (Kranck, 1980). The amount of flocculation in a suspension is dependent on the concentration of particles, the probability that particles adhere when they collide (stickiness), and turbulence, which to a certain level increases flocculation by increasing encounter rate between particles but beyond which it can disrupt flocs (Milligan & Hill, 1998; Winterwerp, 2002; Winterwerp et al., 2006). The result when plotted as log concentration vs log diameter is a DIGS distribution consisting of a straight line, unsorted region, and sorted, modal peaks (Fig. 1) (Kranck & Milligan, 1985, 1991).

The floc model of Kranck et al., (1996a, 1996b) as modified by Curran et al. (2004) assumes that inorganic sediment is deposited within flocs that preserve the size distribution of the parent suspension, or as single grains (Fig. 1). Floc fraction (FF) and floc limit (d_f_), the diameter at which the contribution to the bed sediment from flocs and single grains are equal, vary with the amount of floc deposition and incorporation of inorganic particles into the bottom sediment. Using FF and d_f_ avoids the need to determine the properties of the flocs in situ. DIGS can be used to infer both the amount of material that was deposited in flocs and the degree of flocculation in the overlying water from which it was deposited (Kranck, 1984; Law et al., 2013, 2019; Milligan et al., 2007; Poirier et al., 2017). Milligan and Law (2005) used FF to show that floc deposition increased with the development of open pen salmon aquaculture in Letang Inlet, New Brunswick, Canada. This increase in floc-settled material coincided with an increase in organic material and trace metals (Smith et al., 2005; Yeats et al., 2005) and a change in faunal assemblages in the estuary (Wildish & Pohle, 2005). It is this change in the deposition of fine-grained sediment with associated organic material and possible contaminants that is of concern in aquaculture.

DIGS analysis within DFO has historically been carried out using a Coulter Counter, the most recent instruments being the Beckman Coulter MS3 and MS4 (Kranck & Milligan, 1979; Milligan & Kranck, 1991). The need for lower cost and increased numbers of analyses has made laser diffraction an attractive, although untested, alternative. Typically, laser diffraction can analyze 3–4 times as many samples as a Coulter Counter in a day. The LS has a nominal size range from 0.375 to 2026 µm and the normal operation of the CC using three apertures has a size range from 1 to 296 µm. The LS was originally developed for artificial particles, and it is known that “for non-spherical particles the resulting particle size distribution is different from that obtained by methods based on other physical principles (e.g., sedimentation, sieving)” (ISO13320:^2020^).

Adoption of laser diffraction for DIGS analysis within the large-scale AMP requires that it can accurately measure changes in the environment relevant to aquaculture monitoring in both the near and far field, i.e., is a Beckman Coulter LS 13 320 able to determine if the amount of material being deposited in the form of flocs is changing? As previous comparison studies of the Coulter Counter and laser diffraction have not been definitive, over 500 bottom sediment samples from different depositional environments in eastern Canada were analyzed using either a MS3 or MS4 model Coulter Counter (CC) and a Beckman Coulter 13 320 laser diffraction particle size analyzer (LS) to determine if the LS should be used in the AMP.

## Methods

### Sample collection

**Fig. 2:**
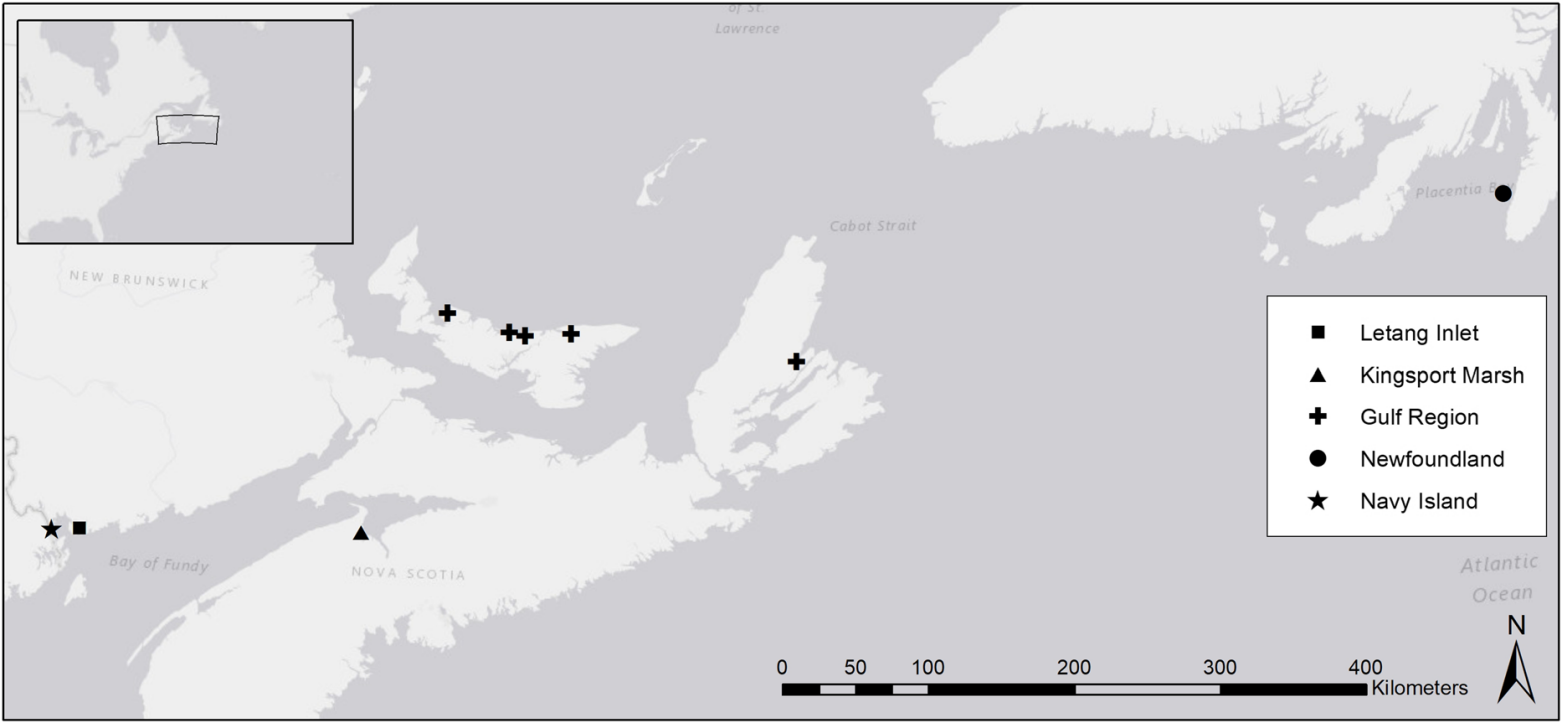
Location map of sampling locations for comparison of results from the CC and the LS

Surficial sediment samples were collected from five areas in eastern Canada (Fig. 2). Three hundred and eight samples were collected from three bays with open pen salmon aquaculture, Letang Inlet, New Brunswick (Letang), Navy Island New Brunswick (Navy), and Placentia Bay Newfoundland (NFLD). As one of the focuses of the AMP is environmental interactions in the far field, samples were collected at the operational site and throughout the bay. One hundred and twenty samples were collected from five different bays used for shellfish aquaculture in the Southern Gulf of St Lawrence (Gulf). Samples were collected throughout the four bays on Prince Edward Island and from one on Cape Breton Island, NS. To evaluate the performance of the instruments in an area with no influence from aquaculture, 153 samples were collected from a macro tidal flat at Kingsport Nova Scotia in the Minas Basin (Kingsport). For Letang, Navy, and NFLD samples, the top 1 cm was taken from either an Eckman or van Veen grab. For Gulf samples, the top 2 cm from cores collected by divers was used. At Kingsport, the top 1 cm of sediment was collected at low tide using a cutoff syringe at fixed locations on the tidal flat. The Letang stations were sampled in March and November 2019 and October 2020. On the Kingsport tidal flat, samples were collected in June and October 2019 and September 2020. The other locations were sampled on one occasion only. All subsamples were run on both the CC and LS.

Samples of Sillikers clay, a fine-grained glacial marine deposit from Sillikers, New Brunswick, used as a lab standard for grain size analysis, were also analyzed on the CC and the LS (Kranck, 1980; Milligan & Hill, 1998; White et al., 2022). Sillikers clay is composed mainly of irregular clay and silt-sized (< 63 µm) kaolinite and illite with < 0.13% carbon (Kranck, 1980; White et al., 2022).

### Sample analysis

Analysis on the CC followed the method described in Milligan and Kranck (1991). In short, sediment samples were dried at < 60 °C and then treated with an excess of 35% H_2_O_2_ to remove organics. The inorganic residue was suspended in a particle-free 1% NaCl solution and disaggregated using a sapphire-tipped ultrasonic probe just prior to counting. A sapphire-tipped horn is used to prevent the addition of particles that can occur due to the deterioration of standard titanium horn tips (Milligan & Kranck, 1991; Rendigs & Commeau, 1987). If required, suspensions were diluted with 1% NaCl to prevent > 5% coincidence, more than one particle present in the sensing zone at the same time. At this concentration of particles, and the short time between sonification and counting, re-aggregation of the particles is negligible (Milligan & Kranck, 1991; White et al., 2022). Three aperture tubes, 30 µm, 200 µm, and 400 µm were used to obtain a continuous size distribution from 1 to 296 µm.

For the Beckman Coulter LS 13 320, subsamples received the same peroxide treatment as stated above for the CC method and were suspended in ultrapure water. They were disaggregated with ultrasonics immediately before analysis using the standard operating procedure for an LS 13 320 equipped with the Aqueous Liquid Module for sample handling (Beckman-Coulter, 2011). At the concentration where obscuration levels are at or below the required level, the re-aggregation of inorganic particles suspended in reagent grade water is much less than the time required to complete the analysis. Size distributions were determined based on the Fraunhofer theory of light scattering (see the detailed protocol from the Beckman Coulter LS 13 320 manual).

The Sillikers clay samples were not pre-treated with H_2_O_2_ because Sillikers clay has a very low organic content. Samples were sonified immediately before analysis. Sample preparation has been shown to have a very minimal effect on the size distribution of Sillikers clay when measured with either the CC or the LS (White et al., 2022).

### Modification of size distributions

Two methods were used to modify the DIGS distribution of samples to evaluate the response of the CC and LS to changing size ranges. In the first, the still water settling experiment of Kranck (1980) using Sillikers clay was duplicated. In an unflocculated suspension, particles will settle in accordance with Stokes law, with the largest particles settling first followed by each smaller size class. This forms the basis of particle size analysis by pipet. In a flocculated suspension, all size classes are removed in the same proportions as they occur in the suspension (Kranck, 1980). The goal was to determine if the LS was able to differentiate these two modes of particle settling. Equal amounts of Sillikers clay were suspended in two cylinders containing either a 3% NaCl (flocculating) or 6% NaPO_3_ (dispersant) solution and allowed to settle after disaggregation using ultrasonics. Twenty-five-milliliter samples were withdrawn from the suspension by pipet at 15 cm depth at 0, 22, 180, and 360 min. These samples were disaggregated with an ultrasonic probe immediately before analysis on the CC and LS.

In a second experiment to examine how the CC and LS respond to decreasing the maximum particle size being analyzed, ten samples from Letang Inlet were analyzed on the CC and LS and then screened at 63 and 16 µm. After analysis of the full-size distribution, subsamples of the suspension were screened to remove particles greater than the nominal screen size. The suspension that passed through each screen was then sonified just prior to analysis on the CC and the LS.

The CC and the LS analyze DIGS over different size ranges, 1–296 µm and 0.04–2026 µm, respectively. For compatibility, particles < 1 µm were deleted for the LS. The extended size range of the LS makes it sensitive to the presence of rare large particles. Because size classes are logarithmic in both instruments, a few large particles can skew the total volume. As the deposition of fine-grained sediment is the focus of this study, the effect of large particles on the fine fraction was reduced by deleting size classes > 194 µm and > 63 µm then renormalizing the remaining size classes to 100% (White et al., 2022).

## Results

**Fig. 3:**
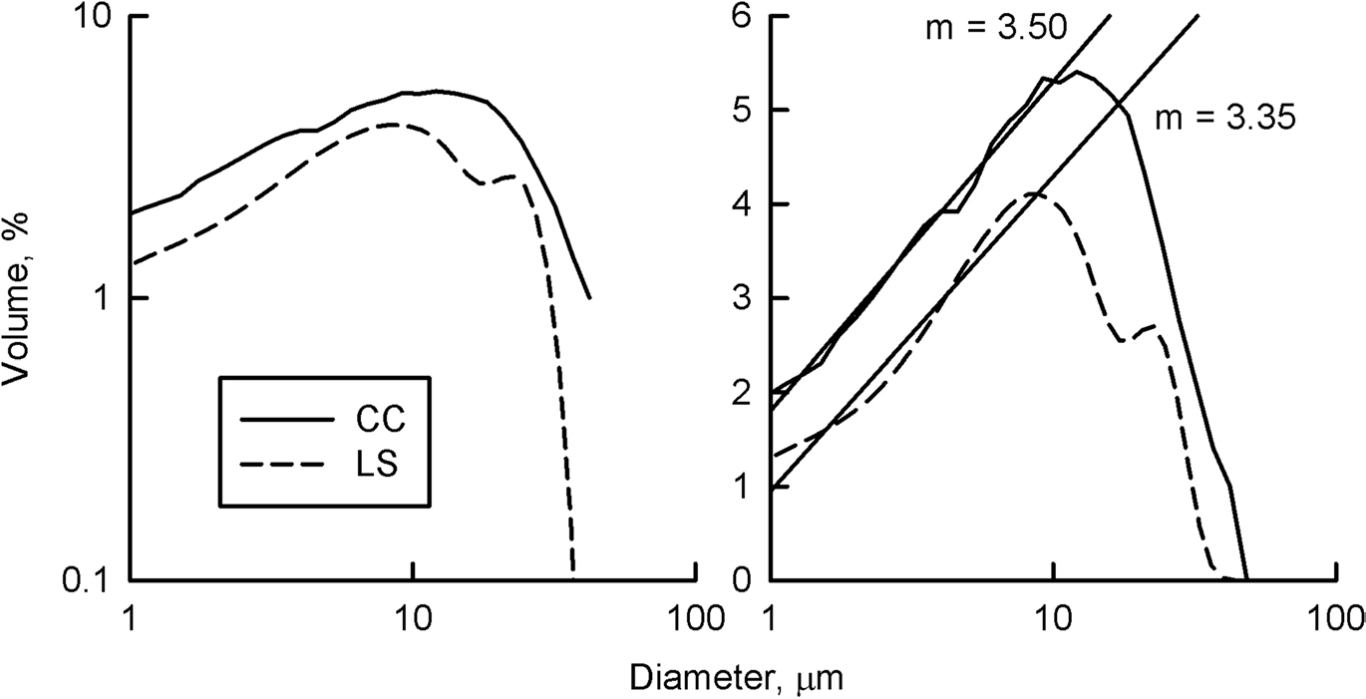
DIGS of Sillikers clay analyzed with a Beckman-Coulter MS3 and LS 13 320 laser diffraction particle size analyzer. Both log log and semi-log plots of % volume are presented to emphasize the difference between the size distributions The LS has two smooth modal peaks where the CC consists of a straight-line portion, single modal peak, and fall off in the large size classes. Lines represent the least squares fit for the semi-log data

The shape of the DIGS distributions from the LS differs from the CC for Sillikers clay in that where the CC has the straight-line distribution in the small size classes predicted by Krumbein and Tisdel (1940) and Kranck and Milligan (1985), the LS produces a primary smooth modal peak centered around 10 µm and a second modal peak at 20 µm (Fig. 3). A linear fit of the < 10-µm fraction illustrates the difference between the interpolation of particle size in the LS and the measurement of individual particles in the CC (Fig. 3). The slope of the curve from the LS is constantly and regularly changing where the CC data is scattered about the line. Smooth modal peaks like those for Sillikers occur in all the samples run on the LS. The floc model of Kranck and Milligan (1985) assumes the presence of an unsorted, straight-line size distribution in the small-size classes which represents the particles settled in flocs.

The DIGS distributions from the Gulf were fitted with the Curran et al. (2004) model to assess if the LS could be used to determine floc fraction. Values for both FF and d_f_ from the LS were less than half those for the CC and standard deviations and variance for both values were much larger for the CC (Table 1). Given the wide range of depositional conditions sampled, d_f_ and floc fraction would be expected to vary considerably (Hill et al., 2007; Law et al., 2019; Milligan et al., 2007).

**Table 1:** Summary statistics for results from fitting the Curran et al. (2004) model to the DIGS from 120 Gulf samples

	CC df (µm)	LS df (µm)	CC FF	LS FF
Mean	23.73	3.89	0.66	0.24
Standard error	1.23	0.11	0.01	0
Standard deviation	13.49	1.22	0.16	0.05
Sample variance	182	1.49	0.03	0
Range	53	8	0.74	0.33

Law et al. (2008) identified 16 µm as the size below which sediment moves cohesively, as part of flocs, but d_f_ and FF values from the LS represent size distributions well below this value and do not vary. Because the LS sees modal peaks within the region of unsorted sediment, the floc model of Curran et al. (2004) cannot be applied. In its place, % sortable silt (%SS) and the volume fraction < 16 µm (% < 16) were used to compare DIGS from the two instruments.

%SS was proposed by McCave et al. (1995) and McCave and Hall (2006) to determine paleo-ocean current speed in the deep ocean based on the assumption that particles > 10 µm can be sorted but particles < 10 microns are winnowed away in proportions equal to their presence in the sediment. The < 10 µm fraction moves as flocs so all size classes below ~ 10 µm are mobilized at the same time (Law et al., 2008; McCave et al., 1995). Sortable silt % has been used to study flocculation in near-shore sediments, where biological effects on floc formation can dominate and bottom stress is high (Chang et al., 2007); Molinaroli et al. (2009); Flemming et al. (2024). Sortable silt % is calculated as follows:

1 $$SS\%=\frac{\sum_{i=10\mu m}^{i=63\mu m}\left(f_i\right)}{\sum_{i=1\mu m}^{i=63\mu m}\left(f_i\right)}\ast100$$

where *f*_*i*_ is the normalized volume concentration in bin *i* (McCave & Andrews, 2019; White et al., 2022). Electro resistance (Bianchi et al., 1999) and laser diffraction instruments (McCave et al., 1986a, 1986b, 2006) have been previously evaluated for determining sortable silts used for estimating deep ocean currents.

As small clay grains must be incorporated in flocs to settle, an increase in the % volume < 16 µm would indicate increased floc deposition (Law et al., 2008). % < 16 is the sum of the volume percentages < 16 µm calculated as follows:

2 $$\%<16={\sum }_{i=1\mu m}^{i=16\mu m}\left({f}_{i}\right)$$

**Fig. 4:**
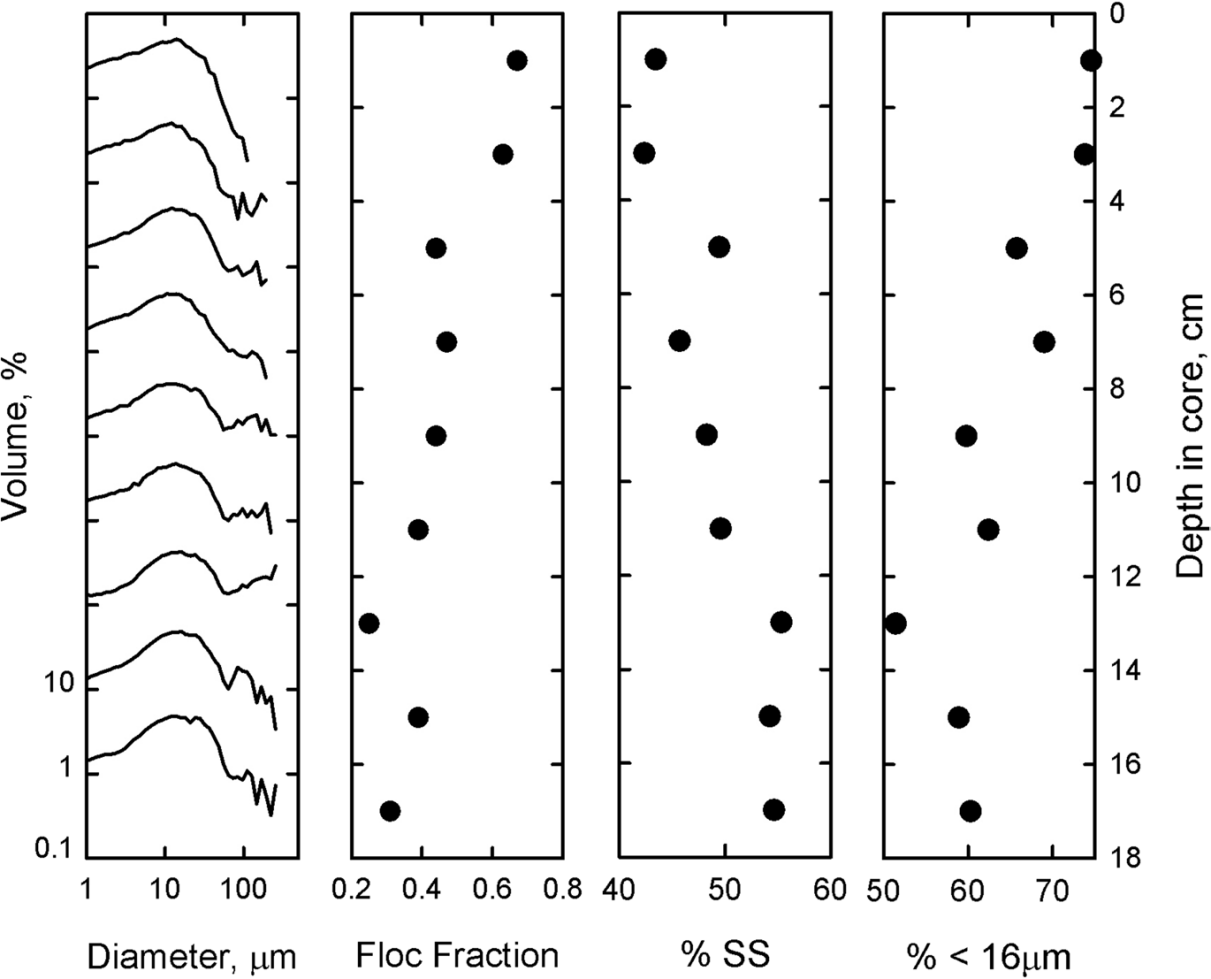
Disaggregated inorganic grain size distributions and values for floc fraction, %SS, and % < 16 from a core taken in Letang Inlet, NB, analyzed with a Coulter Counter. Log log plots of DIGS (left) are vertically offset by 1 decade to show equivalent depth in the core

%SS and % < 16 are unable to determine floc fraction, but both respond to variations in fine particle sedimentation and can be used as a proxy. Like floc fraction, %SS and % < 16 calculated using DIGS from a core analyzed with the CC increase after the introduction of open cage aquaculture in the Letang Inlet as described in Milligan and Law (2005) (Fig. 4).

The %SS and % < 16 data were evaluated for correlation to indicate if both instruments showed similar spatial or temporal trends. The Shapiro-Wilks test was used to test for normality. The null hypothesis of normality was rejected so Kendall’s tau was used to test for correlation. Kendall’s tau was determined for the three size ranges in MatLab^©^. To evaluate if %SS and % < 16 from the CC and LS were statistically similar, a paired *t*-test was run in MatLab^©^, A significance level of 0.05 was used for all tests.

A reduced major axis regression (RMA) was used to illustrate the relationship between %SS and % < 16 from the CC and LS using a MatLab^©^ script (Trujillo-Ortiz et al., 2024). RMA regression was used because there is no dependence between the two measurements and both measurements are subject to error (Miller and Khan, 1962).

**Fig. 5:**
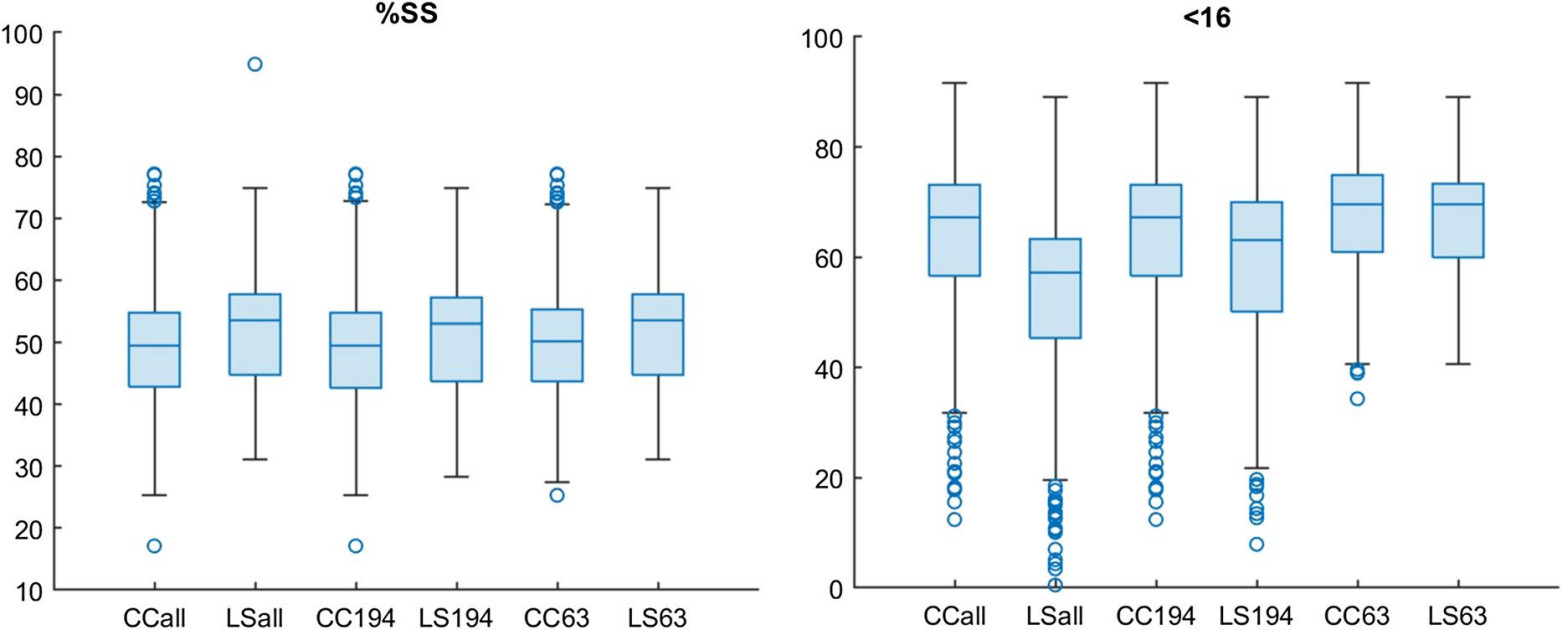
Box and whisker plots of %SS and % < 16 showing the effect of varying the data range. All = 1–2026 µm, 194 = 1–194 µm, and 63 = 1–63 µm for CC and LS data. Values were normalized to 100% for each size range

Reducing the size range over which the data was normalized led to a reduction in the scatter in the % < 16, brought the mean values for the CC and LS closer and reduced the standard deviation (Table 2; Fig. 5). The values for %SS were unaffected by the size range over which the data were normalized because it does not change the ratio of 1–10 µm to 1–63 µm.

**Table 2:** Values for Kendall’s tau correlation for individual areas sampled and for the entire dataset

		1–63 µm		1–194 µm		1–2000 µm	
% < 16	*n*	tau	*p*	tau	*p*	tau	*p*
Letang	204	0.32	< 0.001	0.32	< 0.001	0.31	< 0.001
Kingsport	153	0.41	< 0.001	0.43	< 0.001	0.40	< 0.001
Gulf	120	0.60	< 0.001	0.62	< 0.001	0.56	< 0.001
NFLD	59	0.62	< 0.001	0.57	< 0.001	0.52	< 0.001
Navy	45	0.42	< 0.001	0.43	< 0.001	0.45	< 0.001
All	581	0.64	< 0.001	0.64	< 0.001	0.60	< 0.001
%SS							
Letang	204	0.31	< 0.001	0.30	< 0.001	0.31	< 0.001
Kingsport	153	0.40	< 0.001	0.41	< 0.001	0.40	< 0.001
Gulf	120	0.56	< 0.001	0.58	< 0.001	0.56	< 0.001
NFLD	59	0.52	< 0.001	0.52	< 0.001	0.52	< 0.001
Navy	45	0.46	< 0.001	0.46	< 0.001	0.45	< 0.001
All	581	0.65	< 0.001	0.66	< 0.001	0.65	< 0.001

There is a statistically significant correlation between the two instruments over all normalized size ranges (Table 2) but the correlation is not consistent among sample locations. For example, when normalized from 1 to 63 µm, tau values for the % < 16 fraction and %SS in Letang were 0.32 (*p* < 0.001) and 0.31 (*p* < 0.001), respectively compared to 0.64 (*p* < 0.001) and 0.65 (*p* < 0.001) for the entire data set (Table 1). Results from the paired *t*-test indicate that, apart from NFLD and Navy, the two areas with the smallest number of samples, the null hypothesis that the data are statistically similar was not supported (Table 3).

**Table 3:** Paired *t*-test results for individual areas sampled and for entire dataset showing results for different size ranges. h0 = 1 rejects the null hypothesis that the two population means are equal. SD is the estimated population standard deviation

		1–63 µm			1–194 µm			1–2000 µm		
% < 16	*n*	h0	*p*	SD	h0	*p*	SD	h0	*p*	SD
Letang	204	1	< 0.001	3.97	1	< 0.001	8.91	1	< 0.001	10.62
Kingsport	153	1	< 0.001	5.73	1	< 0.001	6.77	1	< 0.001	6.77
Gulf	120	1	< 0.001	6.85	1	< 0.001	10.45	1	< 0.001	11.92
NFLD	59	1	2.36E − 02	3.52	0	7.57E − 01	5.01	0	7.57E − 01	5.01
Navy	45	1	< 0.001	2.29	0	3.77E − 01	2.47	0	3.79E − 01	2.47
All	581	1	2.08E − 02	5.63	1	< 0.001	8.49	1	< 0.001	10.35
%SS										
Letang	204	1	< 0.001	4.02	1	< 0.001	4.81	1	< 0.001	5.34
Kingsport	153	1	2.03E − 03	4.92	1	< 0.001	5.12	1	< 0.001	5.02
Gulf	120	1	< 0.001	6.56	1	2.21E − 03	6.31	1	< 0.001	6.56
NFLD	59	1	< 0.001	4.14	1	< 0.001	4.14	1	< 0.001	4.14
Navy	45	1	< 0.001	2.20	1	< 0.001	2.24	1	< 0.001	2.25
All	581	1	< 0.001	4.97	1	< 0.001	5.17	1	< 0.001	5.39

**Fig. 6:**
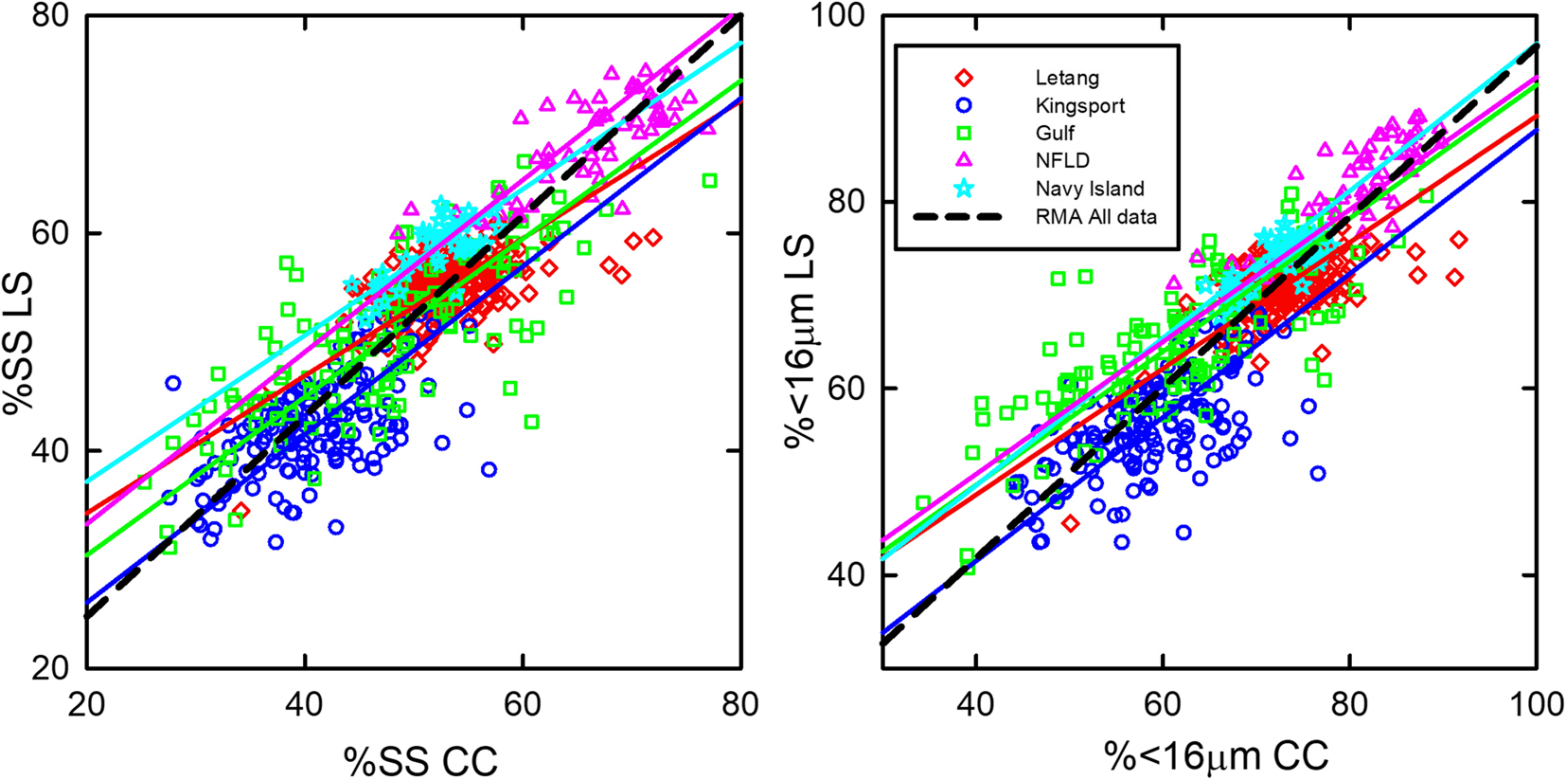
Reduced major axis regressions for %SS and % < 16 µm calculated using the 1–63 µm size range for individual areas and complete data set (see Table 4)

RMA regression for each area and the entire dataset illustrate that the relationship between the CC and the LS is area dependent (Fig. 6). While samples from each of the different depositional environments group together, the values for the slope, intercept, and standard error vary based on the area sampled for both % < 16 and %SS (Table 4). The RMA regression of the entire data set has a much higher slope and lower intercept that does not match any of the areas sampled.

**Table 4:** Reduced major axis regression results for individual areas sampled and for entire dataset showing results for different size ranges

		1–63 µm			1–194 µm			1–2000 µm		
% < 16	*n*	Slope	Intercept	Error	Slope	Intercept	Error	Slope	Intercept	Error
Letang	204	0.68	21.55	5.20	1.15	− 15.86	10.10	1.38	− 39.86	11.61
Kingsport	153	0.77	10.72	6.57	1.00	− 7.63	7.76	0.92	− 7.18	7.73
Gulf	120	0.71	21.09	6.75	0.93	− 0.23	10.89	0.98	− 7.61	12.48
NFLD	59	0.79	18.05	6.10	1.04	− 3.61	8.18	1.04	− 3.61	8.18
Navy	45	0.71	22.44	6.58	0.80	14.79	7.10	0.80	14.79	7.10
All	581	0.92	5.17	5.74	1.10	− 10.94	8.56	1.19	− 21.98	10.24
%SS										
Letang	204	0.63	21.64	4.47	0.57	24.67	4.95	0.75	16.13	6.08
Kingsport	153	0.77	10.58	5.33	0.75	11.74	5.37	0.75	12.03	5.34
Gulf	120	0.73	15.85	6.45	0.76	13.20	6.30	0.73	15.85	6.45
NFLD	59	0.67	23.73	5.84	0.67	23.73	5.84	0.67	23.73	5.84
Navy	45	0.79	17.45	4.89	0.80	17.20	4.91	0.78	18.29	4.90
All	581	0.92	6.34	5.01	0.90	7.14	5.16	0.91	7.31	5.42

## Discussion

Figure 3 shows the DIGS for Sillikers clay. The slope of the CC size distribution is constant from 1 to 10 µm and then concentration regularly decreases with increasing diameter, defining the maximum particle size. In contrast, the LS has two smooth modal peaks and constantly changing slopes. The presence of multiple modal peaks, with values evenly distributed about the mode, is a feature found in all the DIGS samples analyzed in this study. Because it evenly distributes particle volume about a central tendency the LS does not resolve the flat portion of the size distribution with slope *m* (Fig. 1). This results in very similar values for d_f_ and FF being returned over a wide range of depositional environments that the CC discriminates between. The model of Curran et al. (2004) cannot be used with LS data to determine d_f_ and FF for use in monitoring changes in the amount of floc deposition.

The size distribution of particles determined using different methods for the same sample may not be directly comparable, but they should still be valid according to a theoretical relationship. While it is reasonable to expect the CC and LS to measure particle size differently, the two instruments would need to be consistently different to inform changes between locations and over time within a monitoring program. Moreover, the relationship between the LS and CC for parameters like % SS and % < 16 µm, which describe the size distribution between 1 and 63 µm, should not vary based on the location being sampled.

Correlation analysis of %SS and < 16 µm values from the LS and the CC indicate that the two instruments show the same trends overall, but the extent of the correlation differs among sites (Fig. 6). Results from the paired *t*-test imply that the two instruments do not provide the same estimates for %SS and % < 16. Differences between the two instruments are not large but the apparent dependence of the estimates on the area being sampled is of concern if areas that are being developed for aquaculture are to be compared. To better understand the source of this discrepancy, we need to examine in greater detail how the two instruments respond to variations in the DIGS from different environments.

**Fig. 7:**
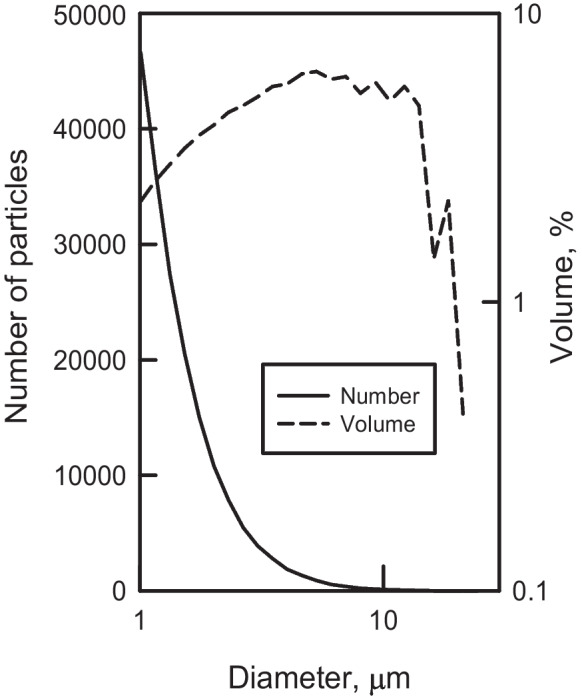
Particle number and particle volume plots for Sillikers clay analyzed with the 30-µm aperture on a Beckman Coulter MS3

The CC measures individual particles whereas the LS interprets the pattern of light scattered by particles using a proprietary manufacturer’s algorithm. Coulter Counters can achieve the size resolution of a mixed solution of mono-sized particles to less than 5%, but laser diffraction can only achieve peak-to-peak resolution of 25–50% (Beckman Coulter, 2016). Figure 7 shows the number and volume distributions of particles for Sillikers clay from a MS3 using the 30-µm aperture. Kowalenko and Burbain (2013) concluded “that the geometric change in a number of particles as the size decreases in a given weight of sample is too large to allow the use of current laser diffraction instrumentation for particle analyses of soil and related samples where the sizes in the distribution are from clay to sand or even with a narrower range of sizes (e.g., clay or silt)”. Low resolution of mixed particle sizes, with exponentially increasing numbers of particles in the smallest size classes leads to the creation of smooth modal peaks in laser diffraction analysis of fine-grained inorganic sediment. The extended size range of the LS can skew the size distribution to the larger classes, especially when there is a low particle number in the largest size classes, leading to overestimation of the size of large particles by as much as 20% (Blott & Pye, 2006). Sperazza et al. (2004) found that with the Malvern Mastersizer® laser diffraction instrument, varying the absorption could lead to bimodality in the size distribution and significantly skew the fine-grained tail of coarser samples. Nonspherical particles with large aspect ratios can produce “… oversized size distribution and exaggerated distribution broadness due to both particle orientation effects and deviation from the spherical models used in data processing” (Xu & Guida, 2003).

**Fig. 8:**
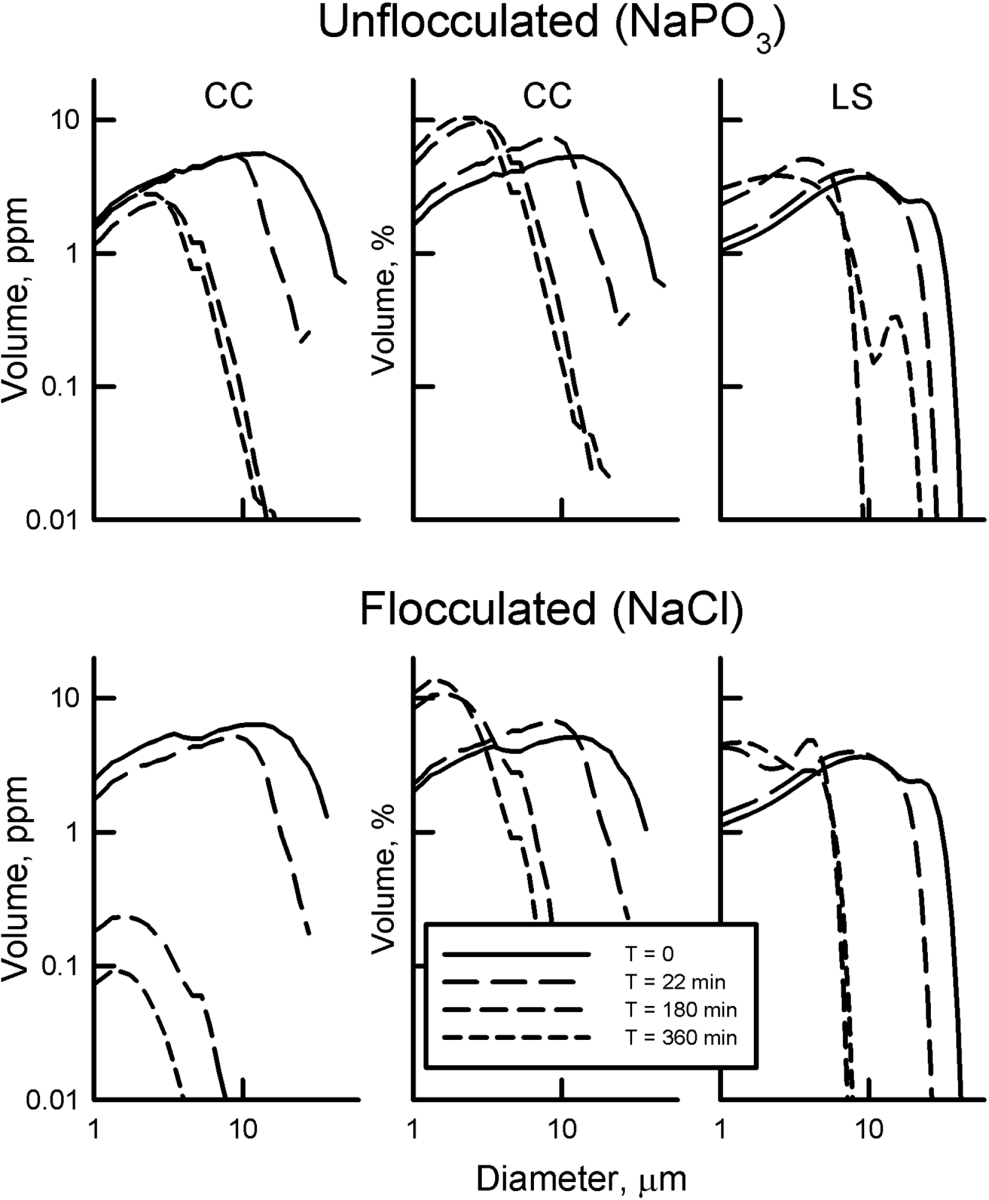
Log log DIGS plots for pipet samples taken during still water settling of unflocculated and flocculated Sillikers clay as described in Kranck (1980). DIGS for each pipet draw off was determined with a Beckman-Coulter MS3 (CC) and Beckman-Coulter LS 13 320. Results from the CC are expressed as volume particles per volume sample (ppm) (left) and as % volume

Similarly, Kowalenko and Babuin (2013) varied the amount of soil of different size classes in an experiment and found that differences in the numbers of particles in the same weight of samples containing particles from sand to clay sizes are too large for laser diffraction instruments because of threshold, saturation, and interaction issues. The LS appears to have difficulty interpolating the diffraction pattern as the relative contribution from different size classes varies during still water settling of Sillikers clay (Fig. 8). In the unflocculated Sillikers suspension, the DIGS from the CC show the largest particles settling first while the concentration in the smallest sizes remains unchanged. With the LS the distributions of the smallest sizes remaining in suspension vary at each time step, and new modal peaks appear as the apparent volume is distributed about a perceived mode (Fig. 8). Floc settling removes particles from a suspension in the same proportions in which they initially occur in the suspension (Kranck, 1980). DIGS from the CC for the flocculated suspension maintains the same unsorted size distribution of the parent suspension at a lower concentration at each time step. The LS again modifies the size distribution in the smallest classes and creates a new modal peak (Fig. 8). There is no physical reason for the variation of particle sorting in the suspension seen by the LS.

**Fig. 9:**
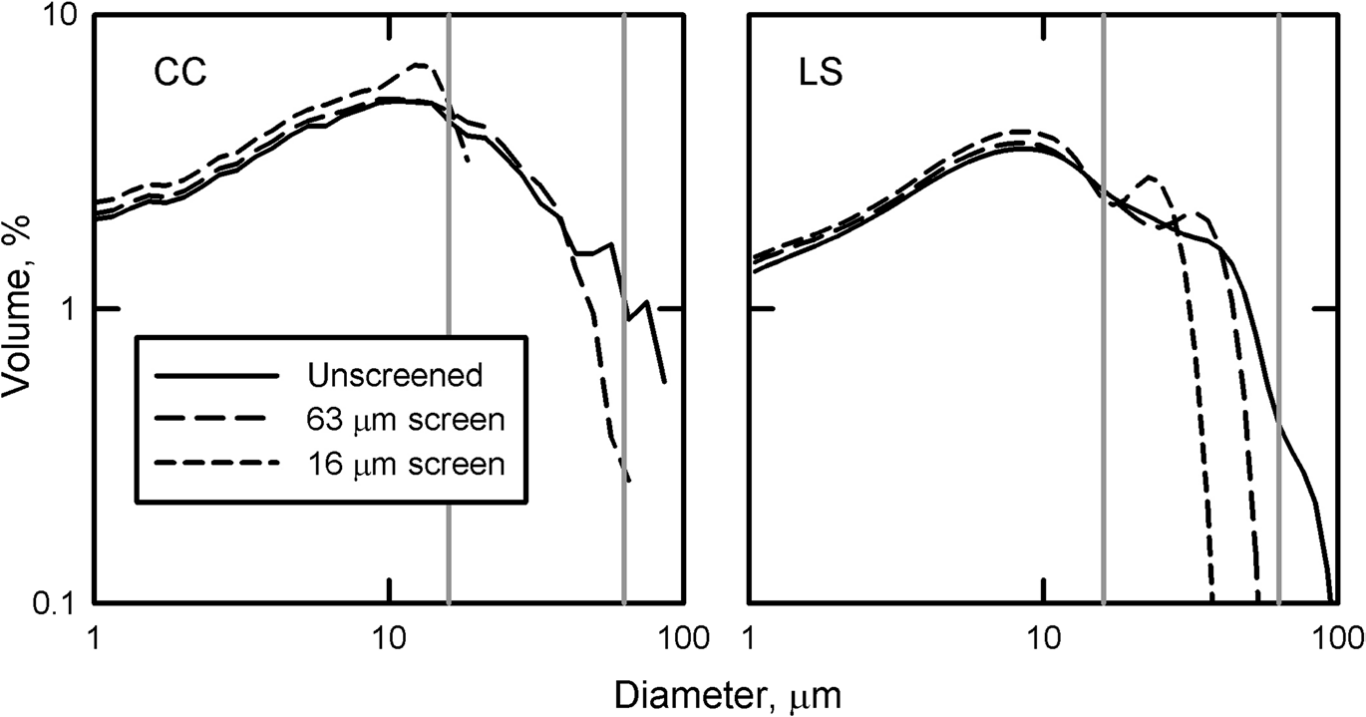
Log log size distributions for one of ten similar samples from Letang Inlet analyzed with a CC (right) and LS (left). Samples were analyzed without screening and after passing through 63 and 16 µm screens (gray vertical lines)

When sediment from Letang Inlet is screened at 63 and 16 µm, the size distributions from the CC maintain the same size distribution up to the nominal screen size but the LS appears to add particles after each screening (Fig. 9). Where the CC shows that there are no particles > 16 µm after screening, the LS has a well-defined modal peak at ~ 20 µm. This peak is of similar shape but smaller diameter to the one that appears after screening at 63 µm. There is no physical reason for the appearance of a new peak larger than the nominal size of the screen.

**Fig. 10:**
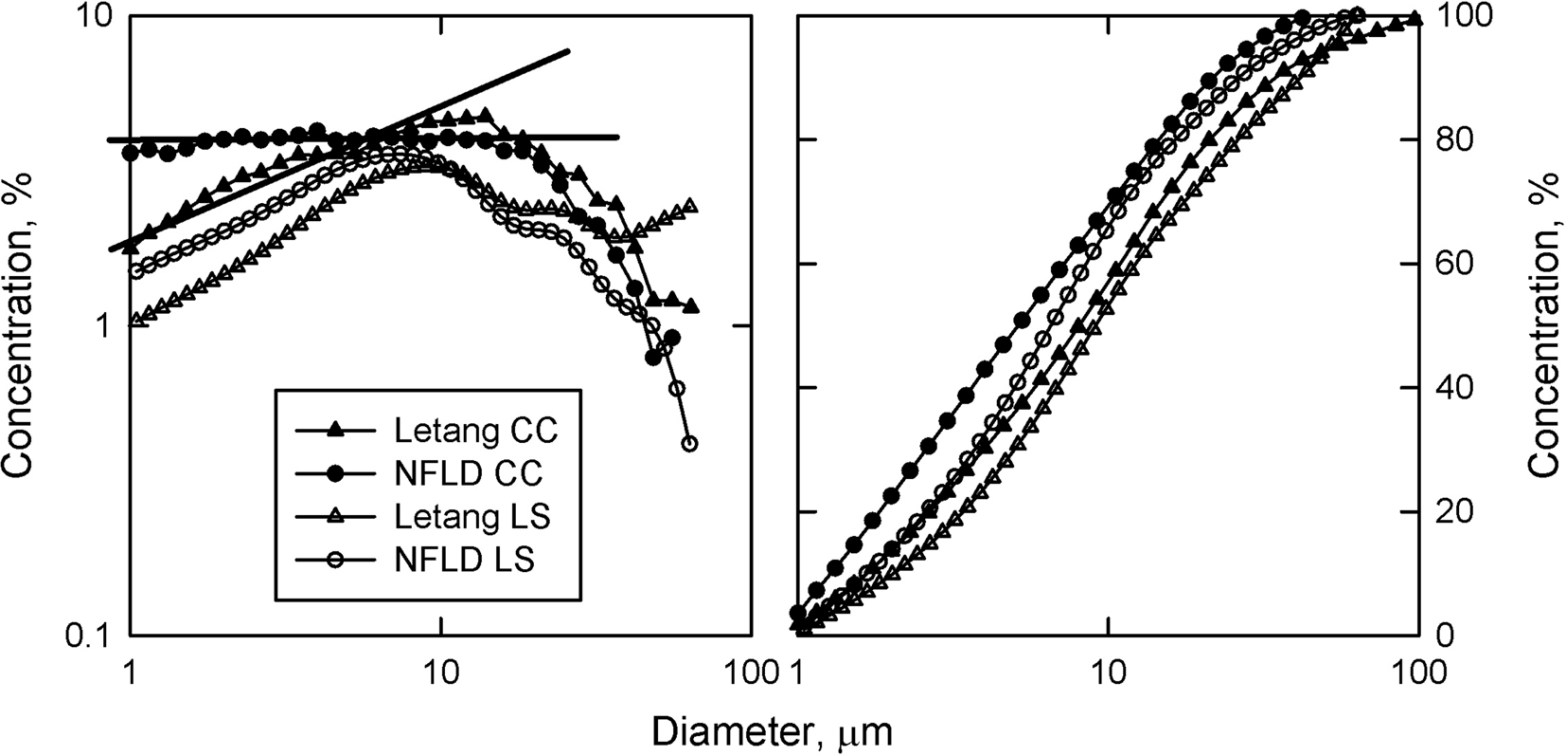
Log log and cumulative size distributions for a sample from Letang Inlet NB and Placentia Bay NFLD analyzed with a CC and a LS. Solid lines in the log log plot are linear regression fits of the CC data for diameters between 1 and 10 µm

The slope of the DIGS from weathered rock varies with the type of rock being eroded (Krumbein and Tisdel, 1940; Kranck & Milligan, 1985). Sediment in Letang Inlet and Placentia Bay (NFLD) is derived from different source rocks and when plotted as log concentration vs log diameter the linear regression of the < 10 µm fraction from the CC shows a distinct difference in the slope of the line compared to the LS, 0.44 and 0.03 respectively. This unsorted, floc-settled potion of the size distribution agrees with Krumbein and Tisdel (1940) and Kranck and Milligan (1985). Distributions for these two samples from the LS have the same shape in both log log and cumulative plots (Fig. 10). In the same manner as the Sillikers clay sample, where the CC finds no sorting, the LS distributes the interpolated volume evenly about central tendency creating a modal peak.

We attribute this difference to the way that the LS analyzes fine-grained, inorganic sediment with unsorted size distributions. Inorganic sediment from low energy aquatic environments, mud, is made up of nonspherical clays with potentially a wide range of indices of refraction leading to “… the formation of modal peaks that center around the general tendency of light intensities caused by particle scattering” reported by Xu and Di Guida (2003). This is due to deposition in these environments being dependent on flocculation, which is an unbiased sampler of the source suspension (Kranck, 1980). Fine particles in suspension resulting from weathering are unsorted, the size distributions of which are dependent on the type of rock from which they are eroded (Krumbein and Tisdel, 1940; Kranck & Milligan, 1985). These broad distributions, consisting of an exponentially increasing number of particles with decreasing size, nonspherical particles, occasional coarse particles, and a range of indices of refraction are the most difficult for the LS to analyse yet they are the most common size distributions in marine sediments. By measuring individual particles, the CC returns the actual distribution of particle sizes where the LS returns a generalized interpolation of the distribution about modal trends.

Based on our results, estimates from the LS will capture variations in the deposition of inorganic fine-grained sediment but the LS is unable to differentiate the DIGS among areas with different sediment sources. Due to the nature of sediment from low-energy aquatic environments, where the CC shows no sorting in the fine particles < 16 µm, the LS invariably sees broad modal peaks which are insensitive to changes in the sediment source. These peaks cause an underestimation of particle area in the smallest size classes. This inability to resolve the size distribution of the inorganic particles in flocs reduces its effectiveness for modelling the transport and deposition of fine particles and surface-active contaminants.

## Conclusion

The goal of monitoring DIGS at aquaculture sites is to quantify changes in the accumulation of fine-grained inorganic particles over time and space to better understand and scale potential near and far field aquaculture impacts on benthic habitat. The DIGS of over 500 bottom sediment samples from a range of depositional environments were analyzed using a CC and a LS. Both instruments can quantify %SS and % < 16 using DIGS but data from the two instruments cannot be used interchangeably and the relationship between the CC and LS varies with the area being sampled. This appears to be due to the difficulty in analysing size distributions when particles are nonspherical, have varying indices of refractions, and are present over a wide range of sizes: which describes a typical bottom sediment from a muddy environment. In this study, we have used the Fraunhofer model of light scattering which is most effective for coarse particles and loses accuracy in particle sizes < 10 µm. Mie theory is proposed for spherical particles < 10 µm, but it requires the correct optical model for the minerals being analyzed. Determination of the mineralogy and optical properties of samples would be beyond the ability of a large monitoring program of different depositional environments with different source sediment. The impact on %SS and % < 16 µm is minimized as these values are derived from a wide range of particle sizes. The inability of the LS to differentiate DIGS among areas with different sediment sources means that for aquaculture monitoring, the LS is limited to monitoring individual areas and should not be used to compare different regions.

The model of Curran et al. (2004) cannot be used with LS data to study the transport and deposition of flocs using bottom sediment DIGS. The ubiquitous, multiple smooth modal peaks seen in the DIGS from all the samples analyzed with the LS do not differentiate areas with different sediment sources. The LS interpolates the size distribution of all sediment as one or more modal peaks with volume evenly distributed about them. The apparent creation of modal peaks, whether particles are present during settling and screening or not, is of concern and should be further investigated. Based on this study, the LS should not replace the CC to monitor and assess the environmental impacts of aquaculture in the AMP.

## Data Availability

All data are in the public domain and available through Fisheries and Oceans Canada. This study is part of the QA QC process being carried out in the development of an online database for aquaculture monitoring in Canada.
